# Comparative experimental study on two designed intravenous anaesthetic combinations in dogs

**DOI:** 10.17179/excli2017-298

**Published:** 2017-05-22

**Authors:** Abdelnaser Abdelmoneim Azab Abdel-Hady, Khaled M. Abdelbasset, Ahmed S. Soliman

**Affiliations:** 1 Department of Surgery, Anaesthesiology and Radiology, Faculty of Veterinary Medicine, South Valley University, Qena, Egypt; 2 Department of Surgery, Anaesthesiology and Radiology, Faculty of Veterinary Medicine, Cairo University, Cairo, Egypt

**Keywords:** propofol, diazepam, xylazine, anaesthesia, dogs

## Abstract

The goal of the present study is to design a good anaesthetic program for dogs which can lead to optimal anaesthesia with no or minimal post-operative adverse effects. For this purpose, we designed two anaesthetic combinations and compared their effects in Mongrel dogs: combination 'A' consisting of atropine, xylazine, ketamine plus propofol, and combination 'B' consisting of atropine, diazepam, ketamine plus propofol. The onset and duration of anaesthesia induction, the duration of maintenance as well as the period of recovery were recorded and compared for both combinations. Furthermore, heart rate, respiratory rate, body temperature as well as blood picture were analyzed before and after administration of the proposed anaesthetic regimens. Administration of combination 'A' lead to rapid onset, within seconds, and induction of anaesthesia. The anaesthetic effect was maintained for approximately 29 minutes after injection of propofol. This was followed by very smooth recovery within approximately 68 minutes after induction of anaesthesia. In contrast, a less efficient induction and maintenance of anaesthesia as well as individual variability was observed after administration of combination 'B'. Approximately 14 minutes were needed for anaesthesia induction, which was maintained for 23 minutes after injection of propofol. Furthermore, administration of combination 'B' was associated with alterations in heart rate, body temperature and hematological picture. In conclusion, our results revealed that combined administration of atropine, xylazine, ketamine plus propofol (combination 'A') is considered satisfactory for anaesthesia in dogs with minimal post-operative side effects.

## Introduction

Anaesthesia practice is a combination of technical skills, experience, compassion and science. Anaesthesia can be classified according to the type of drug used and/or the route of administration (Thurmon and Short, 2007[31]). Basically there are two major ways to obtain anaesthesia in veterinary medicine: either via parenteral injection of anaesthetic drugs (subcutaneously, intramuscularly or intravenously) or via inhalation of volatile anaesthetic agents (Thurmon and Short, 2007[31]). The use of inhalation anaesthetics is not well established in developing countries because of instruments costs and the potential hazard from high pressure tanks. Therefore, parenteral administration of anaesthetics considered the method of choice, specifically, intravenous anaesthetic drugs and techniques which are considered the primary means of chemical restraint in veterinary practice (Amal et al., 2015[1]). However, the design of an optimal combination of pre-anaesthetic and anaesthetic drugs is challenging particularly for pet animals. Thiopental has been considered the sole anaesthetic agent used by veterinarians for most surgical interventions (Muhammad et al., 2009[23]). However, the limited availability stimulates the search for further alternatives. Ketamine is a dissociative anaesthetic and can produce a profound analgesia (White et al., 1980[34]; Clarke and Trim, 2014[5]). However, the use of ketamine alone has two disadvantages: (1) poor muscle relaxation and muscle tone is often increased (Hall et al., 2001[13]), and (2) it produces short-term anaesthesia which is not optimal for long surgical operations (Seliskar et al., 2007[27]). Therefore, it would be ideal to use ketamine in combination with some additives that help to overcome these limitations. Diazepam is a typical anti-convulsion's benzodiazepine derivative (Hall et al., 2001[13]). It can produce muscle relaxation with minimal side effects (Clarke and Trim, 2014[5]). Propofol is a unique non-barbiturate, non-steroid, short-acting general anaesthetic agent that can produce rapid smooth anaesthesia induction and rapid recovery (Hofmeister et al., 2008[15]). The anaesthetic duration of propofol could be enhanced when used in combination with ketamine hydrochloride (Van Natta and Rex, 2006[32]). The drug has been previously used in equines and produced rapid onset of action, short duration of anaesthesia induction and prompt recovery. Studies of combined propofol and alpha 2-agonist as xylazine or detomidine (Branson and Gross, 1994[4]) or benzodiazepine (Guit et al., 1990[12]) or ketamine (Minoru et al., 2004[21]) showed an additive anaesthetic effect. Premedication with either xylazine or detomidine improved the quality of anaesthesia produced by a single bolus of propofol (Mathews et al., 1999[20]). 

The goal of the present study is to test the anaesthetic effect of two selected drug combinations in dogs in order to identify the ideal cocktail which leads to optimal anaesthesia and minimal post-operative adverse effects.

## Materials and Methods

### Animals

The study was performed in 10 Mongrel dogs which were randomly divided into two groups, five animals each. All experiments were approved by the local animal ethics committee. The animals were kept under the same managemental and nutritional conditions during the anaesthetic trials. Food was withheld for 12 hours and water for 2 hours prior to induction of anaesthesia to avoid vomiting and respiratory disorders. The dogs were casted on lateral recumbancy, with head slightly lower than the hind quarters. Respiratory rate, pulse rate and temperature of each dog were recorded prior to the experiment.

### Chemicals

The chemicals which were used in this study are summarized in Table 1(Tab. 1).

### Experimental design

**Figure 1:**
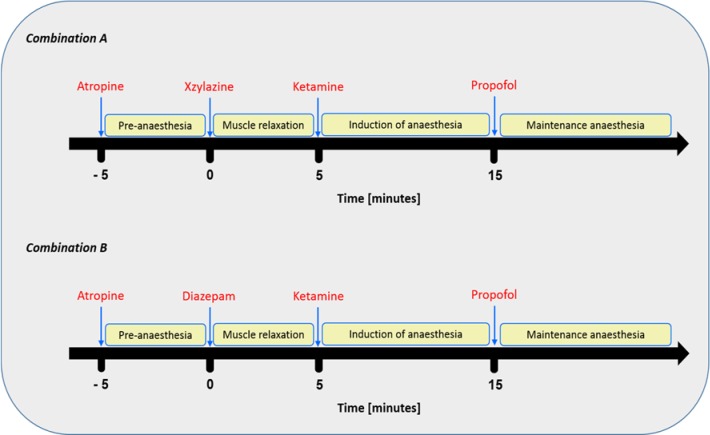
Schedules of composition and administration time of the anaesthetic combinations

The site of injection was clipped, shaved and disinfected. Subsequently, a 20 gauge catheter was inserted in the cephalic vein. The anaesthetic compounds were administered in order as illustrated in Table 2(Tab. 2) and Figure 1(Fig. 1).

### Haematological analysis

For measurement of blood picture, blood samples were collected in tubes pre-coated with heparin to avoid blood coagulation. Subsequently, complete blood picture was analyzed using automatic blood cell counter (Erma PCE 210 N, Japan). 

### Heart rate, respiratory rate, and body temperature

The heart rate was measured via auscultation according to Ko et al. (2006[16]). The respiratory rate was determined by visual observation of the thoracic motion per minute (Lemke et al., 2002[18]). The rectal temperature was measured using a clinical thermometer.

## Results

### Evaluation of the anaesthetic efficiency of the designed drug combinations

Administration of combination 'A' (atropine, xylazine, ketamine and propofol) as illustrated in Figure 1(Fig. 1) led to rapid onset, within a minute after ketamine injection, and induction of anaesthesia in all dogs. The anaesthesia was maintained for approximately 29 minutes following propofol administration (Table 3(Tab. 3)). A very smooth recovery and unaided standing was reached within 68 minutes following the induction of anaesthesia. Combination 'B' (atropine, diazepam, ketamine and propofol) was less efficient both in induction as well as maintaining of anaesthesia in comparison to combination 'A'. In addition, there was variability on the onset of anaesthesia; three dogs showed rapid and smooth onset within a minute after ketamine administration. Whereas, mild (less alert but still active) to moderate (drowsy, recumbent) sedation was observed in other animals. However, induction of anaesthesia in all animals occurred within approximately 14 minutes following ketamine injection and maintained for approximately 23 minutes following the administration of a maintenance dose of propofol (Table 3(Tab. 3)). A period of approximately 53 minutes was required for unaided standing and smooth recovery.

Following xylazine, ketamine (combination 'A') injection, all dogs showed deep sedation; very drowsy and unable to walk. During the premedication and the induction phases, all body reflexes were very sluggish and the eyeballs were centrally located. Injection of ketamine leads to deep plane anaesthesia with good degree of muscle relaxation and downward eyeball. Whereas, after propofol administration deep anaesthesia was maintained with very good muscle relaxation (Table 4(Tab. 4)). Following injection of diazepam, ketamine (combination 'B'), all dogs showed moderate to deep sedation (very drowsy, recumbent and unable to walk) with complete analgesia in 4 dogs and moderate analgesia in one animal. After induction of anaesthesia and during the maintenance period complete analgesia was recorded in all dogs. With regard to the depth of anaesthesia, anal, pedal and palpebral reflexes showed a slight depression in all dogs following administration of the premedication of combination 'B'. While the swallowing and pupillary reflexes appeared normal. The eyeballs were ventromedially located only in three dogs. Good muscle relaxation was recorded in all dogs. After injection of ketamine, deep plane of anaesthesia was achieved with good degree of muscle relaxation. Following the administration of propofol, deep anaesthesia was maintained with very good muscle relaxation only in two dogs and good relaxation in the other three dogs (Table 4(Tab. 4)).

### Analysis of the possible adverse effects following administration of the designed drug combinations

In order to check whether there side effects develop following administration of the drug combinations the following parameters were analyzed:

### Heart rate, respiratory rate and body temperature

Administration of combination 'A' did not lead to any significant alteration in heart or respiratory rates as well as in the body temperature (Table 5(Tab. 5)). Following administration of combination 'B' there was no significant changes in the respiratory rate. In contrast, a slight increase in the heart rate was recorded directly after propofol administration (Table 5(Tab. 5)). In addition, the body temperature showed non-significant decrease following induction of anaesthesia, but after injection of propofol, the body temperature decreased significantly and remained low along the observation period (Table 5(Tab. 5)).

### Blood picture

There were non-significant changes in red blood cells (RBCs) count and hemoglobin (Hb) concentration following administration of combination 'A'. However, a significant increase in lymphocytes, monocytes and blood platelets was recorded. After administration of combination 'B', an increase on RBCs count and Hb concentration was recorded. In addition, an initial elevation (during the first 15 min) followed by suppression of lymphocytes count was recorded. Whereas, blood platelets as well as monocytes showed elevation at all tested time periods (Tables 6(Tab. 6) and 7(Tab. 7)).

## Discussion

An ideal balanced anaesthesia produces sleep, amnesia, analgesia and muscle relaxation with no or minimal adverse effects (Morton, 1989[22]). In order to induce anaesthesia in dogs, sedative and analgesic drugs should be given prior to administration of the anaesthetic compounds (Enouri et al., 2008[9]). The use of these pre-anaesthetics possesses several advantages in terms of decreasing anxiety, reducing the dose of the anaesthetic drugs, providing analgesia during the subsequent surgery or diagnostic procedure and leads to smooth induction and recovery from anaesthesia (Enouri et al., 2008[9]). In the present study, we designed two-anaesthetic combinations and compared their anaesthetic efficacy in Mongrel dogs, aiming at reaching optimal anaesthesia with minimal post-operative side effects. Intravenous injection of combination 'A' (atropine, xyalzine, ketamine and propofol) leads to rapid onset and induction of anaesthesia which was maintained for approximately 29 minutes following propfol administration. This was associated with very good muscle relaxation during the entire anaesthetic period. The use of ketamine alone is already known to possess disadvantages including increased muscular tone and fast recovery of animals (Hall et al., 2001[13]; Seliskar et al., 2007[27]). The present results revealed that the additives which were used here in combination with ketamine allowed overcoming these limitations. Sinclair (2003[29]) reported that, using α2-adrenoceptor agonist, e.g. xylazine, in combination with ketamine leads to sedation, analgesia and muscle relaxation in dogs. Furthermore, Waelbers et al. (2009[33]), Short and Bulfalari (1999[28]) and Stoelting (1999[30]) reported that, combined administration of propofol and ketamine helps to prolong the period of surgical tolerance; in addition to the sedative and hypnotic properties. Approximately 68 minutes after anaesthesia induction all dogs showed very smooth recovery, without any apparent undesired effects. These results are in agreement with Muhammad et al. (2009[23]); Andreoni and Lynne Hughes (2009[2]) and Dyson (2007[8]). However, it should be considered that the time of recovery may depend on the dose of propofol.

In contrast to combination 'A', combined administration of atropine, diazepam, ketamine and propofol (combination 'B') was less efficient in terms of anaesthesia induction and maintenance and also showed individual variability. The induction of anaesthesia required approximately 14 minutes after ketamine injection and maintained for approximately 23 minutes after propofol administration. These results are in agreement with Hall et al. (2001[13]) and Rankin (2002[25]) who reported that the sedative and hypnotic effects of diazepam are minimal or absent in dogs. Another reason why diazepam is not used to control seizure in dogs is that the body develops tolerance very quickly. In most dogs, tolerance is observed within a week of starting diazepam treatment (Boothe, 2016[3]).

In order to check the possible adverse effects following administration of both combinations, heart rate, respiratory rate and body temperature were measured before as well as at different stages after drug injections. After administration of combination 'A', there were no significant changes in these parameters. However, several reports showed that propofol injection is associated with dose-dependent respiratory depression (Sano et al., 2003[26]; Covey-Crump and Murison, 2008[6]). Other studies reported that combined administration of ketamine and propofol helps to prevent the respiratory disorders associated with propofol injection alone (Muhammed et al., 2009[23]). In contrast, after administration of combination 'B' the heart rate and the body temperature were significantly compromised. The significant increase in heart rate might be due to panic produced by diazepam as a result of muscle relaxation which was already suggested by Marntell and Nyman (1996[19]), Koshy et al. (2003[17]) and Davison et al. (2007[7]). Furthermore, administration of combination 'B' was associated with compromised blood picture. Plumb and Pharm (1999[24]) reported that propylene glycol in diazepam formulation may cause cardiotoxicity in small animals in case of rapid intravenous injection and sometimes associated with pain during administration. On contrary, Haskins et al. (1986[14]) reported that, when xylazine was administered in combination with ketamine in dogs, this resulted in significantly reduced cardiovascular disturbances. The hematology results revealed no significant alterations after administration of combination 'A'. However, the health state of the animal, the age as well as the dose of the anaesthetic agent may strongly influence the blood parameters (Fayyaz et al 2009[10]; Ferreira et al 2015[11]; Amal et al., 2015[1]).

In conclusion, the present study demonstrates that combined administration of atropine, xylazine, ketamine plus propofol at specified time and doses leads to optimal anaesthesia in dogs with minimal post-operative side effects. 

## Figures and Tables

**Table 1:**
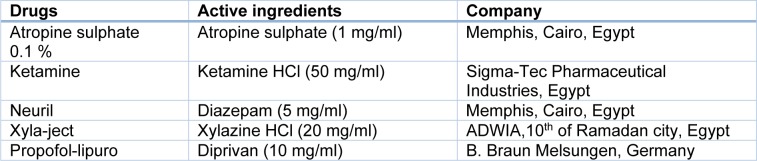
Chemicals

**Table 2:**
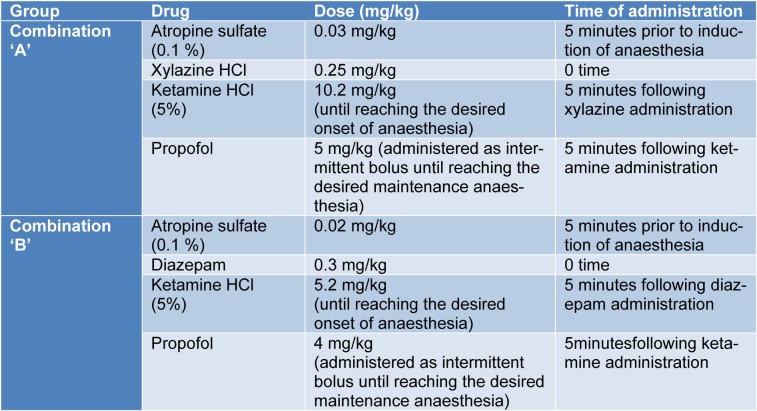
Composition, doses and time of administration of the anaesthetic combinations

**Table 3:**
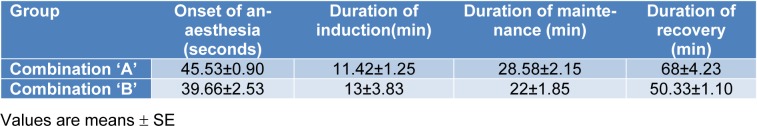
Anaesthetic efficiency of the proposed combinations

**Table 4:**
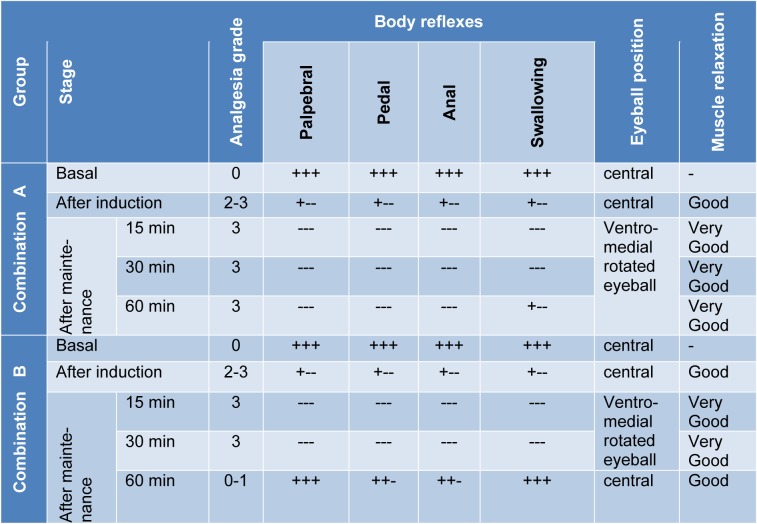
Influence of administration of the proposed drug combinations on sedation, analgesia, body reflexes, eyeball position and muscles

**Table 5:**
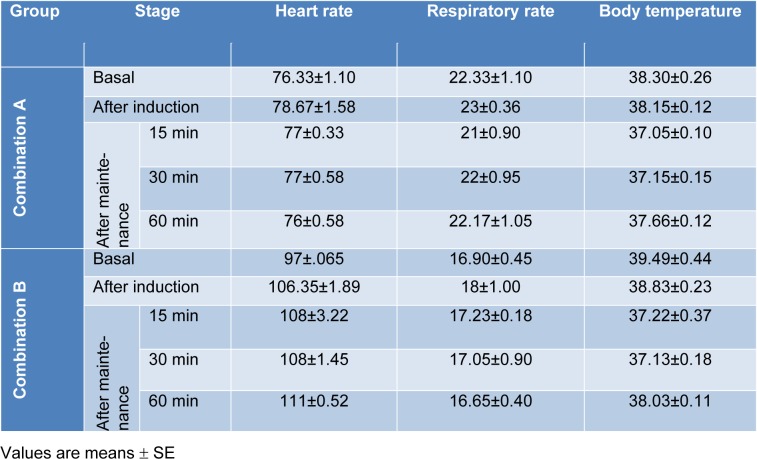
Changes in heart rate, respiratory rate and body temperature following administration of the anaesthetic combinations

**Table 6:**
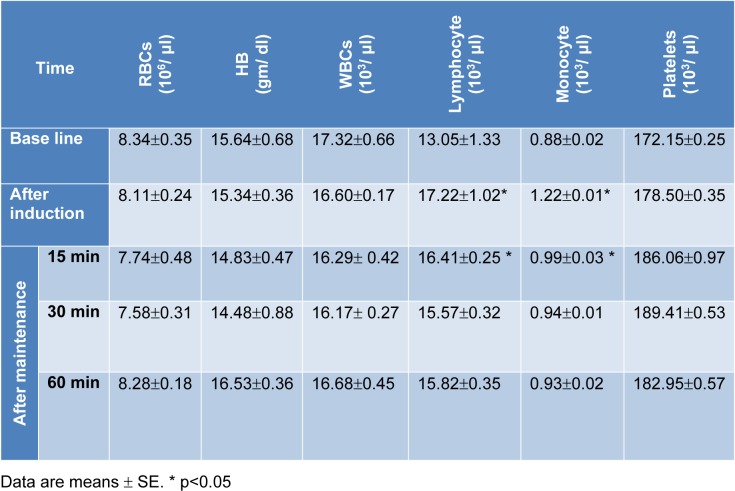
Blood picture before and after administration of combination 'A'

**Table 7:**
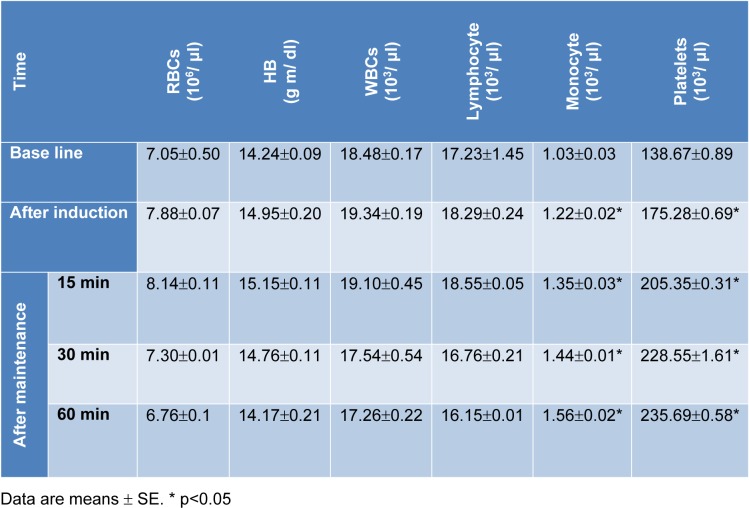
Blood picture before and after administration of combination 'B'
